# A 15-year survey of reproductive efficiency of Standardbred and Finnhorse trotters in Finland - descriptive results

**DOI:** 10.1186/1751-0147-52-40

**Published:** 2010-06-14

**Authors:** Terttu Katila, Tiina Reilas, Kaisa Nivola, Terttu Peltonen, Anna-Maija Virtala

**Affiliations:** 1 Department of Production Animal Medicine, University of Helsinki, Paroninkuja 20, 04920 Saarentaus, Finland; 2 MTT Agrifood Research Finland, Opistontie 10, 32100 Ypäjä, Finland; 3 Kallelan oriasema, Isonummentie 212, 23600 Kalanti, Finland; 4 Suomen Hippos, Tulkinkuja 3, 02650 Espoo, Finland; 5 Department of Veterinary Biosciences, University of Helsinki, P.O. Box 66, 00014 Helsinki, Finland

## Abstract

**Background:**

The major horse breeds in Finland are the Finnhorse (FH) and the American Standardbred (SB). The foaling rates of the FH have consistently been lower than those of the SB. During the last years, a decreasing trend in foaling rates of both breeds has been observed. The purpose of this study was to describe and compare the structure of these two mare populations for age, reproductive history and mating type. In addition, changes over the years were studied that could explain the decline in foaling rates.

**Methods:**

In Finland, the mating statistics and foaling rates per stallion are published yearly by Suomen Hippos, which is the Finnish trotting and breeding association authorized by the EU. The studied material was the electronic breeding data of Suomen Hippos in 1991-2005 which contained 69 180 cases (one mare bred in one year with one stallion), 20 168 mares, 2 230 stallions and 5 397 stud managers. The effect of mare age and type, mating type and changes during the study period were examined separately for FH and SB using SAS 9.1 for descriptive statistical analyses (frequencies, percentages, means, standard deviations and confidence intervals). The outcome of the last mating per season (foal or not) was used in the calculation of the foaling rates.

**Results:**

The FH mares were on average one year older and belonged to the older age groups more often than the SB mares. Ageing decreased foaling rates and even more in FH; the foaling rates were the following: young FH 68.6 and SB 72.1%, middle-aged FH 66.1 and SB 71.9%, ageing FH 61.2 and SB 68.4%, and very old FH 52.8 and SB 61.8%. The foaled mares were more frequent in the SB (45%) than in the FH (37%), but the barren and rested mares were more common in the FH. Natural mating was more commonly practiced in the FH as compared to the SB. The foaling rates decreased from 1991 to 2005 in SB from 75.1 to 65.9% and in FH from 66.5 to 60.8%. For both breeds, the proportion of young mares decreased and the proportion of very old mares increased over the years. Similarly, the proportion of foaled mares in both breeds decreased and the proportion of barren mares increased during the study period. In both breeds, insemination (AI) by transported cooled semen increased, diminishing the on-site AI in the SB and the natural mating in the FH.

**Conclusions:**

The results of this study suggest that mare age and type and mating type all affect foaling rates and that the structural differences in the mare populations can explain differences in the foaling rates between the horse breeds and between the time periods.

## Background

Profitable horse breeding requires general information about the reproductive traits of horses, which horse breeders can then utilize in decision making. However, most research in equine reproduction has focused on individual animals, physiology and pathology. Only one epidemiological study based on a large number of horses (535 746 matings) has been published [1]. This French study comprised several breeds: the Thoroughbred (TB), Anglo-Arab, Selle Francais, French Trotter and the cold-blooded French breeds. An Australian study compared the reproductive efficiency of the TB and the Standardbred (SB) horses [2]. Whereas several reports on TB farms have been published [3-12], less has been published on other breeds: Hannoverians [13], Icelandic horses [14], feral horses [15] and different horse breeds in the USA [16] and in Finland [17].

In Finland, the mating statistics and foaling rates per stallion are published yearly by Suomen Hippos, which is the Finnish trotting and breeding association authorized by the EU. During the recent years, a decreasing trend in foaling rates has been suspected.

Finland has its own horse breed, the Finnish cold blood, the Finnhorse (FH). This breed is of universal type and is mostly used for sports. The average height at withers is 156 cm and the weight is 550 kg. The present population is 19 500 horses from which 200 are breeding stallions and 2 300 brood mares. Furthermore, approximately 1 300 foals are born yearly [18].

Annual reports of Suomen Hippos show higher foaling rates for the SB than for the FH.

The total population of the SB in Finland is about 25 000 making it the largest horse breed in Finland. According to the data base of Suomen Hippos, 64% of the SB mares were born in Finland and 36% were imported. Annually, 90 stallions and 2 600 mares are used for breeding and 1 600 foals are born. In addition, semen from approx. 50 foreign stallions is imported both as cooled and frozen, mainly from Sweden, Italy, Germany and USA. The use of imported semen has increased from 50 to approximately 500 mares per year; half of the mares are inseminated with frozen semen.

The Finnish studbooks and horse registers are kept and administered by Suomen Hippos. To maintain these records, the stallion managers have to report all matings of the breeding season to one of the sixteen local horse breeding associations by the end of October. If the mating has not been reported, the foals are not registered and they are not allowed to race. As a consequence, one can assume that practically all the matings of the trotter mares and births of the foals are reported. Moreover, since 1990, local breeding associations submit the mating records in digital form to the central database of Suomen Hippos.

The objective of this study was to examine the Finnish horse breeding data for possible differences between the two breeds and for changes in the foaling rates through the years. The frequency distributions and foaling rates are reported for each breed, year, time period, age, mare type and mating type. The aim was to seek for any structural changes and differences in the mare populations or mating methods that could explain the possible differences between the breeds and years.

## Methods

The data originates from all the reported inseminations of the FH and SB trotter mares in Finland during the years 1991-2005. For this project, the records in the database of Suomen Hippos were imported to Excel^®^.

One case was defined as one mare bred in one year with one stallion. Only the outcome of the last mating per season (foal or not) was used. The original data consisted of 70 238 cases; 1 002 cases were excluded from the data because the mare type could not be defined. These excluded mares had entered the database in the first years of the digital registration and their foaling history was unknown. Mares over 25 years old (n = 28) were all excluded owing to the small likelihood of their conceiving at this advanced age. One-year-old mares were also excluded (n = 25) because their mating was not planned but accidental, and therefore, only mares which foaled had been reported. Another group excluded from this study was the only three FH mares artificially inseminated (AI) with frozen semen. After these exclusions, the final number of cases was 69 180. It is important to note that the same mares, stallions and stallion managers occur several times because the data covers 15 years.

### Definitions

Foaling rate was used as the measure of reproductive efficiency. In the horse breeding literature, the foaling rate is used for the proportion of mares (%) that have foaled the following year of all mares mated. The Finnish database also includes matings per cycle, but since they had not been consistently reported, the foaling rate was considered to be a more reliable measure.

In the database of Suomen Hippos, the result of the breeding is divided into seven categories. In this study, the categories filly, colt, dead foal and foal died after birth were assigned the value 1 (foaled), and the categories barren, aborted and mare died pregnant were given the value 0 (not foaled).

The change in foaling rates through the years was studied both from year to year and in longer time periods. The 15 years in the study were divided in four time periods (TP): TP1 = 1991 - 1994, TP2 = 1995 - 1998, TP3 = 1999 - 2002 and TP4 = 2003 - 2005.

Mares were divided into four age groups: young (2 - 9 years), middle-aged (10 - 13 years), ageing (14 - 16 years), and very old (17 - 25 years). The different mare types in the data were 1) maiden (never mated), 2) barren (mated earlier, but aborted or did not conceive), 3) rested (not mated after the last foaling), and 4) foaled (mare with a live or dead foal).

The type of breeding was categorised as 1) on-site AI (mare inseminated with fresh semen at the same station where the stallion was resident), 2) AI using transported semen (mare inseminated with cooled semen transported to another station or home stable), 3) AI using frozen semen (only in SB), and 4) natural mating.

### Statistics

After the data had been checked for possible mistakes, typing errors and outliers, they were transported to the SAS 9.1 statistical package (SAS Institute Inc.) for descriptive statistical analyses. The data were described using frequencies, percentages, means, and standard deviations. The 95% binomial confidence intervals for the percentages were calculated using the FREQ procedure.

## Results

There were altogether 69 180 breeding cases and 102 615 cycles, out of which 33 699 breeding cases/49 339 cycles were for the FH mares and 35 481 cases/53 276 cycles for the SB mares. The data included 20 143 mares: 9 633 FH and 10 510 SB. About 30% of the mares appeared in the data more than once. The same FH mare appeared in the data a maximum of 24 times and the same SB mare 18 times. About 3% of the mares (594 mares) had more than 10 breeding seasons.

There were altogether 2 230 stallions (1 201 FH and 1029 SB) and 5 397 stallion managers. Stallion managers were divided according to the breed of the stallion: 3 472 stallion managers had FH stallions and 2 190 SB stallions.

### Mares

The young mares were the largest age group, but whereas they comprised 47% of the SB mare population, they represented only 37% of the FH mares. Correspondingly, the FH occurred more frequently in the very old mare group than the SB (13 versus 8%). The proportions of middle-aged mares were 33% in FH and 32% in SB and the proportions of ageing mares 17 and 13%, respectively. The median ages were 11 years for the FH and 10 years for the SB.

Foaled mares were the largest mare type in both breeds, but more frequent in the SB (45%) than in the FH (37%). On the other hand, barren and rested mares were more common in the FH (26 and 15%) than in the SB (21 and 12%). The proportions of maiden mares were 21% in FH and 22% in SB.

When the age within the mare types was considered, the young SB mares were more frequent in the categories of maiden, barren and foaled mares than the FH; no difference was detected in the rested mares (Table 1). In the middle-aged and ageing groups, the maiden FH mares were more common than the SB mares.

**Table 1:** Proportions (% and 95% CI) of different mare age groups in different mare types for Finnhorses (FH) and Standardbreds (SB) in years 1991 - 2005.

	Maiden		Barren		Rested		Foaled	
	%	95% CI	%	95% CI	%	95% CI	%	95% CI
Young								
FH	**83.2**	82.3 - 84.1	**25.0**	24.1 - 25.9	**26.3**	25.1 - 27.5	**23.8**	23.0 - 24.5
SB	**90.5**	89.8 - 91.1	**35.9**	34.8 - 37.0	**25.6**	24.3 - 26.9	**37.0**	36.3 - 37.8
Middle-aged								
FH	**14.7**	13.9 - 15.5	**34.9**	33.9 - 35.9	**39.2**	37.8 - 40.5	**38.8**	37.9 - 39.6
SB	**8.2**	7.6 - 8.8	**36.7**	35.6 - 37.8	**43.4**	42.0 - 44.9	**39.3**	38.5 - 40.0
Ageing								
FH	**1.7**	1.4 - 2.0	**21.7**	20.9 - 22.6	**20.3**	19.2 - 21.4	**22.0**	21.2 - 22.7
SB	**1.1**	0.8 - 1.3	**15.9**	15.1 - 16.7	**18.7**	17.5 - 19.8	**15.1**	14.6 - 15.7
Very old								
FH	**0.3**	0.2 - 0.5	**18.3**	17.5 - 19.1	**14.3**	13.4 - 15.3	**15.5**	14.8 - 16.1
SB	**0.3**	0.2 - 0.4	**11.6**	10.8 - 12.3	**12.3**	11.3 - 13.3	**8.6**	8.2 - 9.0

n FH	6987		8870		5236		12606	
n SB	7869		7401		4355		15856	

Age groups: Young = 2 - 9 years, Middle-aged = 10 - 13 years, Ageing = 14 - 16 years, Very old = 17 - 25 years.

In the whole material, half of the FH were naturally mated, whereas only one quarter of the SB mares were. In the SB, the on-site AI was the most common mating type (41%), as compared to 28% of the FH mares; natural mating was most common for the FH population. Transported semen was used more frequently to the foaled mares as compared to the other mare types (Table 2).

**Table 2:** Proportions (% and 95% CI) of different mating types in different mare types for Finnhorses (FH) and Standardbreds (SB) in years 1991 - 2005.

	Maiden		Barren		Rested		Foaled	
	%	95% CI	%	95% CI	%	95% CI	%	95% CI
On-site AI								
FH	**29.3**	28.2 - 30.3	**28.1**	27.1 - 29.0	**27.8**	26.6 - 29.0	**27.4**	26.6 - 28.2
SB	**42.4**	41.3 - 43.5	**44.2**	43.1 - 45.3	**40.5**	39.0 - 41.9	**39.8**	39.0 - 40.6
Transported semen								
FH	**19.5**	18.5 - 20.4	**18.0**	17.2 - 18.8	**19.9**	18.8 - 21.0	**27.1**	26.3 - 27.9
SB	**25.5**	24.6 - 26.5	**24.9**	23.9 - 25.9	**26.8**	25.5 - 28.2	**32.9**	32.1 -- 33.6
Frozen semen								
SB	**5.3**	4.8 - 5.8	**4.8**	4.3 - 5.3	**3.7**	3.2 - 4.3	**5.5**	5.1 - 5.9
Natural cover								
FH	**51.3**	50.1 - 52.5	**53.9**	52.9 - 55.0	**52.3**	50.9 - 53.6	**45.5**	44.7 -- 46.4
SB	**26.8**	25.8 - 27.8	**26.1**	25.1 - 27.1	**29.0**	27.6 - 30.3	**21.8**	21.2 - 22.5

n FH	6987		8870		5236		12606	
n SB	7869		7401		4355		15856	

### Changes over the study period

The structure of the Finnish broodmare population changed during the study period. For instance, the proportion of young mares decreased over the years in both breeds (Table 3); the proportion of young mares in the FH group fell from 40 to 30% between 1991 and 2005 and those in the SB group fell from 54 to 41% during the same period. In addition, during the same years, the proportion of the very old mares changed from 11 to 16% in the FH and from 6 to 11% in the SB.

**Table 3:** Proportions (% and 95% CI) of different mare age groups in different time periods for Finnhorses (FH) and Standardbreds (SB).

	1991-1994		1995-1998		1999-2002		2003-2005	
	%	95% CI	%	95% CI	%	95% CI	%	95% CI
Young								
FH	**39.9**	38.9 - 41.0	**41.3**	40.3 - 42.2	**33.8**	32.8 - 34.8	**30.3**	29.2 - 31.4
SB	**53.9**	52.8 - 54.9	**50.6**	49.6 - 51.6	**42.8**	41.8 - 43.8	**40.8**	39.7 - 41.9
Middle-aged								
FH	**31.0**	30.0 - 32.0	**32.3**	31.4 - 33.2	**34.5**	33.5 - 35.5	**33.7**	32.6 - 34.8
SB	**29.8**	28.8 - 30.7	**31.6**	30.6 - 32.5	**34.6**	33.6 - 35.5	**33.6**	32.6 - 34.7
Ageing								
FH	**18.3**	17.5 - 19.1	**14.4**	13.7 - 15.1	**17.9**	17.1 - 18.7	**20.3**	19.4 - 21.3
SB	**10.7**	10.1 - 11.4	**11.1**	10.5 - 11.8	**14.3**	13.6 - 15.0	**14.5**	13.7 - 15.3
Very old								
FH	**10.8**	10.1 - 11.4	**12.0**	11.4 - 12.7	**13.8**	13.1 - 14.5	**15.7**	14.8 - 16.6
SB	**5.7**	5.2 - 6.1	**6.7**	6.2 - 7.2	**8.3**	7.8 - 8.9	**11.1**	10.4 - 11.8

n FH	8313		9917		8791		6678	
n SB	8971		9401		9434		7675	

Age groups: Young = 2 - 9 years, Middle-aged = 10 - 13 years, Ageing = 14 - 16 years, Very old = 17 - 25 years.

Another finding is that over the years, the proportion of foaled mares decreased from 46 to 30% in the FH and from 53 to 39% in the SB. The proportion of barren mares increased in both breeds, but no changes were observed in the maiden mares (Table 4).

**Table 4:** Proportions (% and 95% CI) of different mare types in different time periods for Finnhorses (FH) and Standardbreds (SB).

	1991-1994		1995-1998		1999-2002		2003-2005	
	%	95% CI	%	95% CI	%	95% CI	%	95% CI
Maiden								
FH	**20.1**	19.3 - 21.0	**21.8**	21.0 - 22.6	**19.1**	18.3 - 19.9	**22.1**	21.1 - 23.1
SB	**20.3**	19.5 - 21.2	**25.1**	24.2 - 25.9	**20.5**	19.7 - 21.4	**22.8**	21.9 - 23.8
Barren								
FH	**24.7**	23.8 - 25.6	**24.7**	23.8 - 25.5	**27.3**	26.4 - 28.3	**29.5**	28.4 - 30.6
SB	**18.5**	17.7 - 19.3	**18.9**	18.1 - 19.7	**22.2**	21.3 - 23.0	**24.4**	23.5 - 25.4
Rested								
FH	**9.6**	9.0 - 10.3	**16.0**	15.3 - 16.7	**18.3**	17.4 - 19.1	**18.6**	17.7 - 19.5
SB	**8.1**	7.6 - 8.7	**13.0**	12.3 - 13.6	**14.2**	13.5 - 14.9	**13.9**	13.1 - 14.7
Foaled								
FH	**45.6**	44.5 - 46.6	**37.5**	36.6 - 38.5	**35.3**	34.3 - 36.3	**29.8**	28.7 - 30.9
SB	**53.0**	52.0 - 54.1	**43.1**	42.1 - 44.1	**43.1**	42.1 - 44.1	**38.8**	37.7 - 39.9

n FH	8313		9917		8791		6678	
n SB	8971		9401		9434		7675	

In mating types, the change in favour of insemination with transported semen was quite marked in both breeds (FH from 14 to 31% and SB from 15 to 36%) (Table 5). On-site AI decreased in SB (from 50 to 36%) but stayed the same in FH. The use of natural mating became less popular in both breeds: from 55 to 40% in FH and from 31 to 20% in SB. The use of frozen semen increased slightly (from 4 to 8%) in SB.

**Table 5:** Proportions (% and 95% CI) of different mating types in different time periods for Finnhorses (FH) and Standardbreds (SB).

	1991-1994		1995-1998		1999-2002		2003-2005	
	%	95% CI	%	95% CI	%	95% CI	%	95% CI
On-site AI								
FH	**30.8**	29.8 - 31.8	**25.7**	24.8 - 26.6	**27.2**	26.2 - 28.1	**29.2**	28.1 - 30.3
SB	**50.1**	49.0 - 51.1	**42.6**	41.6 - 43.6	**36.5**	35.5 - 37.5	**35.7**	34.6 - 36.8
Transported semen								
FH	**14.0**	13.2 - 14.7	**19.8**	19.0 - 20.6	**25.4**	24.5 - 26.3	**30.7**	29.6 - 31.8
SB	**15.4**	14.7 - 16.2	**26.4**	25.5 - 27.3	**38.1**	37.1 - 39.1	**36.1**	35.1 - 37.2
Frozen semen								
SB	**3.9**	3.5 - 4.3	**4.7**	4.3 - 5.1	**4.4**	4.0 - 4.8	**7.8**	7.2 - 8.4
Natural cover								
FH	**55.2**	54.2 - 56.3	**54.5**	53.5 - 55.5	**47.4**	46.4 - 48.5	**40.1**	39.0 - 41.3
SB	**30.7**	29.7 - 31.6	**26.3**	25.4 - 27.2	**21.0**	20.1 - 21.8	**20.3**	19.4 - 21.2

n FH	8313		9917		8791		6678	
n SB	8971		9401		9434		7675	

### Foaling rate

**Figure 1:**
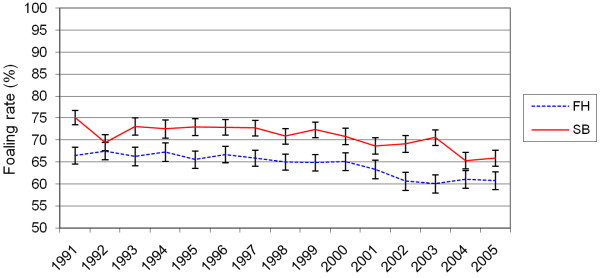
**Foaling rates (mean with 95% confidence interval) in the different years for the Finnhorses (FH) and Standardbreds (SB)**.

**Figure 2:**
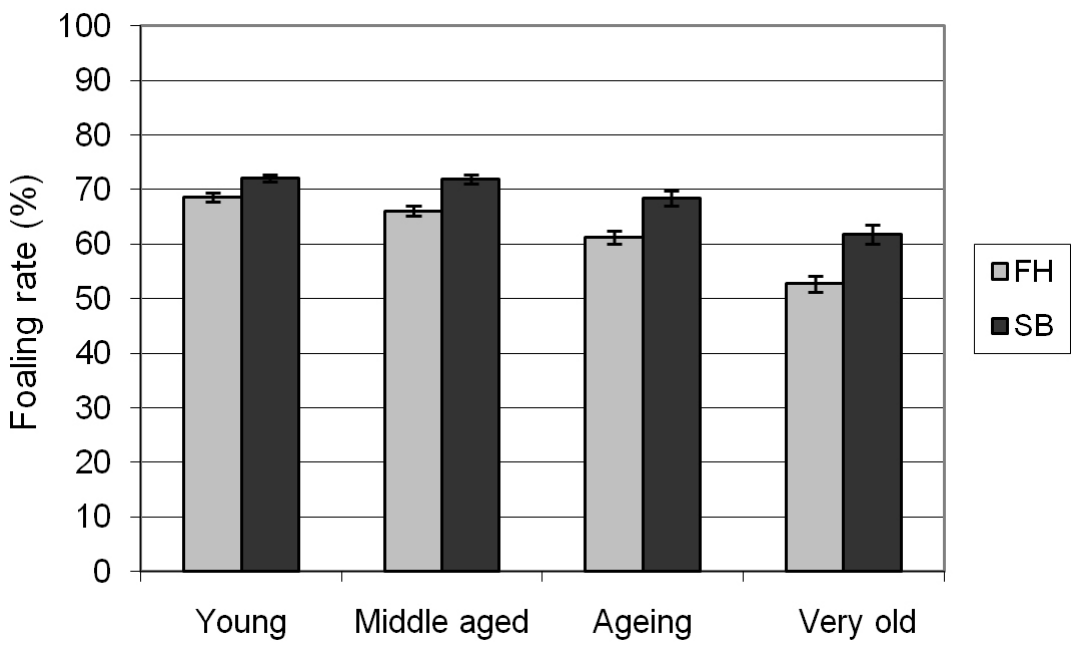
**Foaling rates (mean with 95% confidence interval) in the different age groups for the Finnhorses (FH) and Standardbreds (SB) in the years 1991 - 2005**. Age groups: Young = 2 - 9 years, Middle-aged = 10 - 13 years, Ageing = 14 - 16 years, Very old = 17 - 25 years.

**Figure 3:**
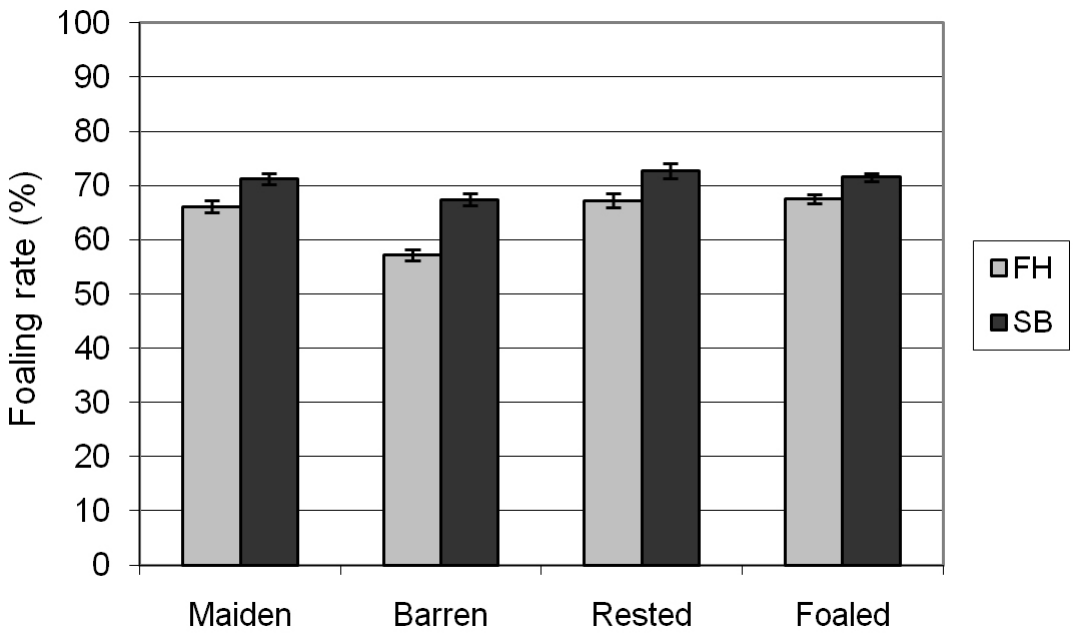
**Foaling rates (mean with 95% confidence interval) in the different mare types for the Finnhorses (FH) and Standardbreds (SB) in the years 1991 - 2005**.

The foaling rate in the whole material was 68%: 64% in the FH and 71% in the SB. There was a lowering trend in the foaling rates from 71% (66% in FH and 75% in SB) in 1991 to 63% (61% in FH and 66% in SB) in 2005 (Figure 1). Foaling rates declined with increasing age in both breeds (Figure 2). Barren mares displayed the lowest foaling rates (Figure 3). In both breeds, the rested and foaled mares in young and middle-aged groups were the most fertile while the very old maidens were the least fertile (Table 6). Moreover, the very old and ageing barren and the ageing maiden FH mares showed low foaling rates.

**Table 6:** Foaling rates in different mare types and age groups for Finnhorses (FH) and Standardbreds (SB) in years 1991 - 2005.

	FH				SB	
	
	n	foaling rate%	95% CI	n	foaling rate %	95% CI
Young	12405			16756		
Maiden		67.9	66.7 - 69.1		71.7	70.6 - 72.7
Barren		63.9	61.9 - 65.9		69.4	67.7 - 71.2
Rested		71.9	69.6 - 74.3		76.4	73.9 -78.9
Foaled		72.1	70.5 - 73.8		72.9	71.8 - 74.1
Middle-aged	11066			11479		
Maiden		59.9	56.9 - 62.9		67.9	64.3 - 71.5
Barren		59.1	57.4 - 60.1		68.7	67.0 - 70.5
Rested		68.9	66.9 - 70.9		73.8	71.9 - 75.8
Foaled		70.7	69.4 - 72.0		73.1	72.0 - 74.2
Ageing	5880			4470		
Maiden		39.7	30.9 - 48.4		63.9	53.5 - 74.2
Barren		54.4	52.2 - 56.6		66.2	63.4 - 68.9
Rested		67.9	65.1 - 70.7		69.8	66.6 - 72.9
Foaled		64.4	62.6 - 66.1		69.3	67.4 - 71.1
Very old	4348			2776		
Maiden		37.5	18.1 - 56.9		52.2	31.8 - 72.6
Barren		47.8	45.4 - 50.2		58.8	55.5 - 62.1
Rested		53	49.4 - 56.6		65.4	61.4 - 69.5
Foaled		57	54.8 - 59.2		62.4	59.8 - 65.0

Age groups: Young = 2 - 9 years, Middle-aged = 10 - 13 years, Ageing = 14 - 16 years, Very old = 17 - 25 years.

**Figure 4:**
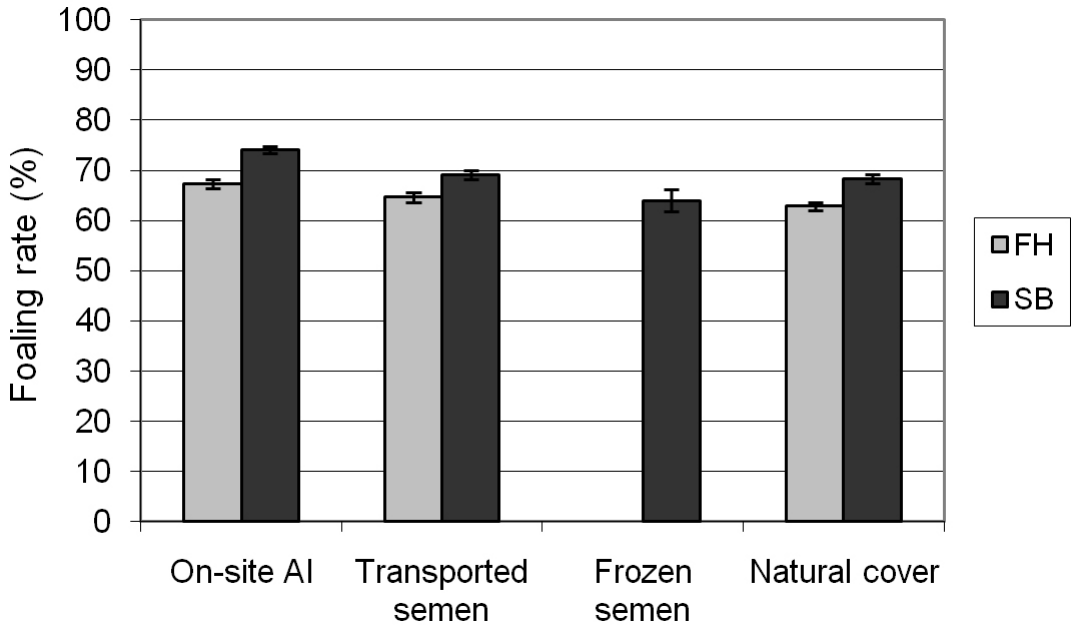
**Foaling rates (mean with 95% confidence interval) in the different mating types for the Finnhorses (FH) and Standardbreds (SB) in the years 1991 - 2005**.

The least successful mating type of the FH was natural mating and the most successful was the on-site AI. For the SB, the lowest foaling rate was found in the frozen semen group and the highest rate in the on-site AI group (Figure 4).

## Discussion

The data analysed showed declining foaling rates during the study period. Moreover, it was very obvious that the foaling rates of the FH were lower than those of the SB.

### Differences between FH and SB

The FH exhibited lower foaling rates than the SB. It is possible that the FH has genetically inferior fertility as compared to the SB, but the differences in the management of the mares and stallions, in both the age structure of the horses and in the proportions of the different mare types, may also explain the difference in foaling rates. Langlois and Blouin [1] reported lower fertility for cold-blooded horses, but pointed out that this may be related to the differences in reporting foals. Management plays an important role: the intensively and professionally managed TB mares have very high pregnancy and foaling rates [9]. In contrast, the FH owners may represent a more traditional horse owner because natural mating was more commonly practiced for the FH than for the SB. Foaling rates after natural mating were lower than after on-site AI which was more common in the SB. The FH mares are probably more often owned and handled by amateurs who might not even sell their foals. The breeding and foal fees are lower for the FH which may also exert an effect on the efforts to get a foal.

The FH mares were on average one year older than the SB mares, which is likely to cause some difference in the foaling outcome. Many studies report the negative effect of ageing of mares on the rates of pregnancy and foaling and on the pregnancy loss [1,3,6,8-12,16,19-21]. It is well established that fertility starts to decrease in middle age: according to Allen et al. > 9 years [11], Laing and Leech > 10 years [3], Hutton and Meacham > 12 years [16], Hemberg et al. > 13 years [10], Morris and Allen, Sanderson and Allen, and Bosh et al. ≥ 14 years [6,9,12], and Hearn et al. > 15 years [8]. In our data, the FH were overrepresented by about 10 percentage points in the two older age classes as compared to the SB. Moreover, the foaling rates in these two higher age groups were lower than in the two younger age groups for both breeds. The FH had foaling rates that were 4-5 percentage points lower than the SB in all age groups, except in the very old mare group where the difference was 9 percentage points higher. Since the FH have lower foaling rates at all ages, age alone may not explain the difference between the breeds, although the higher mean age of the FH certainly contributes to the difference.

Factors that affect a mare's chance of foaling are the age and the reproductive history of the mare. There were less foaled mares in the FH than in the SB. Correspondingly, the barren mare group was larger in the FH than in the SB. Foaled mares are the most fertile group [9], with the exception of the mares bred in foal heat which have lower pregnancy rates [5,9] and higher pregnancy loss rates [9,19] than the mares mated in subsequent heats. Maiden mares are also highly fertile [8,9,11,12]. In contrast, barren mares have the lowest pregnancy rates and the highest pregnancy loss rates [6,8,11,12,19]. In all mare types, the FH had 4-5 percentage points lower foaling rates than the SB, but for barren mares, the difference was 10 percentage points. Although mare type alone may not explain the difference between the FH and SB, it may be a contributing factor to the difference, since the distribution of mares into mare types was less favourable in the FH than in the SB.

The interaction of age and mare type has to be considered as well. Particularly in the maiden mare group, the FH mares were older than those of the SB. Moreover, the old maiden mares have been shown to be the least fertile of all when inseminated with frozen semen [22]. The FH mares seemed to enter the barren mare category at a younger age than the SB mares. In all, the FH mare population differed from the SB population both in terms of age and mare type and age within the mare type. All these differences were unfavourable to the FH and can at least partly explain the lower foaling rates of the FH mares.

### Decreasing foaling rates

The foaling rates showed a decline over the years, which was particularly noticeable since 2000. The decrease in the foaling rate from 1991 to 2005 was over 10 percentage points for the SB, which was greater than for the FH. In fact, the foaling rate difference between the breeds has diminished from the initial 10% to 5%. The foaling rates (for live foal) in TB breeding increased from 1983 to 1998 by 5 percentage points [9] but decreased from 1998 to 2002 by 3 percentage points [9,11]. During the decade 1989-1999 in France, foaling rates increased in all breeds, but more so for the cold-blooded horses, which were less productive to start with; the TB breed progressed very little [1]. However, these studies are from a different time period as the present study and therefore cannot be directly compared. Finland has seen the same trend, with the foaling rates increasing in the 1980 s because of the new reproductive technology, the on-site AI and ultrasonography.

During the follow-up time, the proportion of young mares decreased both in the FH and the SB, and correspondingly the proportions of other age classes increased. Thus, the development in the mares' age has been unfavourable and may contribute to the decline in foaling rates. Furthermore, the proportion of foaled mares diminished (16 percentage points for FH and 14 for SB) and the proportion of barren mares increased in both breeds (5 percentage points for FH and 6 for SB). During this time, the proportion of the rested FH mares doubled and the proportion of these increased in the SB mares by almost 5 percentage points. These represent major changes from the most fertile mare groups to the less fertile mare types.

The structure of the brood mare population for age and reproductive history has not been studied in other breeds with the exception of the TB. Allen and his coworkers published three studies about reproductive efficiency in TB in Newmarket area. These publications contain pregnancy and foaling rates and their analysis from mating seasons 1982-3, 1998 and 2002 [6,9,11]. This allows also us to look at changes over the years and compare the distributions of ages (Table 7) and mare types (Table 8) between TB, SB, and FH. The division of mares into age categories in the English studies was slightly different from ours but the results are still comparable. The time periods were not exactly the same, since the first English study was carried out 10 years earlier than our first time period.

**Table 7:** Age distribution of Thoroughbreds (TB), Standardbreds (SB) and Finnhorses (FH).

Mating seasons	Young (%)	Middle-aged (%)	Ageing (%)	Very old (%)
TB				
1982-3	51.8	30.0	14.6	3.6
1998	53.5	29.7	14.0	2.8
2002	45.1	32.8	17.0	4.3
SB				
1991-4	53.9	29.8	10.7	5.7
1995-8	50.6	31.6	11.1	6.7
1999-2002	42.8	34.6	14.3	8.3
FH				
1991-4	39.9	31.0	18.3	10.8
1995-8	41.3	32.3	14.4	12.0
1999-2002	33.8	34.5	17.9	13.8

Young = 3-8 y for TB and 2-9 y for SB and FH; middle-aged = 9-13 y for TB and 10-13 y for SB and FH; ageing = 14-18 y for TB and 14-16 y for SB and FH; very old = > 18 for TB and 17-25 for SB and FH.

TB data 1982-3 from Sanderson and Allen (1987), 1998 from Morris and Allen (2002), and 2002 from Allen et al. (2007) [6,9,11].

**Table 8:** Distribution of different mare types in Thoroughbreds (TB), Standardbreds (SB) and Finnhorses (FH).

Mating seasons	Maiden (%)	Barren (%)	Foaling (%)	Rested (%)
TB				
1982-3	11.9	24.7	63.4	N.R.
1998	16.2	16.8	65.5	1.5
2002	17.7	24.7	57.6	N.R.
SB				
1991-4	20.3	18.5	55.0	8.1
1995-8	25.1	18.9	43.1	13.0
1999-2002	20.5	22.2	43.1	14.2
FH				
1991-4	20.1	24.7	45.6	9.6
1995-8	21.8	24.7	37.5	16.0
1999-2002	19.1	27.3	35.3	18.3

Barren mare category includes also aborted mares. N.R. = not reported.

TB data 1982-3 from Sanderson and Allen (1987), 1998 from Morris and Allen (2002), and 2002 from Allen et al. (2007) [6,9,11].

The age structure of TB and SB was similar, but FH were much older. While the mares in our material became older, the TB population was younger in 1998 than earlier or later. Maybe this is also the reason for the higher pregnancy and foaling rates reported for the season 1998 as compared to 1982-3 and 2002. However, a clear ageing of the TB mare population from 1982-3 to 2002 was evident similar to our observations.

The proportion of foaling mares throughout these years was approximately 10 percentage points higher in TB than in SB and 20 percentage points higher than in FH (Table 8). The maiden mare population was smaller in TB than in our trotters but the barren mare group was similar in size. It is unclear, why rested mares were lacking in the TB studies from 1982-3 and 2002, but since they comprised only 1.5% in 1998, it can be concluded that they did not represent a large group. This group was much higher in the SB and FH trotters. This may reflect the professionalism in TB breeding: once the mare has started as a brood mare, it is consistently bred each year. In Finland, many breeders have one or two mares which they breed only for their own purposes, not for sale. Economical constraints may also play a role; in Finland the foal prices and selling prospects have sometimes been so low that it is better not to breed the mare again for a year or two.

During the first half of the 1980 s natural mating gave way to the on-site AI in Finland. In the second half of the 1980 s, transported semen started to replace the on-site AI. This development - a decrease in natural mating and in the on-site AI and an increase in transported semen - continued during the study period of 1991-2005. In the FH, the proportion of the on-site AI did not change much, but while the proportion of transported semen doubled, the proportion of natural mating decreased by 15.1 percentage points. Moreover, the proportion of transported semen doubled in the SB, but both the on-site AI (-14 percentage points) and natural mating (-10 percentage points) decreased. In addition, the use of frozen semen increased by 4 percentage points. Almost 44% of the SB mares are inseminated using techniques where the individual stallion differences in the sperm survival during storage can result in a large variation in the foaling rates.

Natural cover - which is the only allowed mating method for the TB - results in very high rates of pregnancy and foaling in the TB industry [9-12], but in other breeds where the AI is allowed and used, higher pregnancy rates have usually been reported for the AI than for the natural mating. For instance, Langlois and Blouin [1] demonstrated the highest foaling rates for the on-site AI. The next best method was free pasture mating (not practiced in Finland); similar results were yielded for transported semen, frozen semen and in-hand natural mating. For both breeds in Finland, the on-site AI was the best method; foaling rates after the use of natural mating and transported semen were similar, but lower as compared to the on-site AI, and frozen semen resulted in the lowest foaling rate. The differences in foaling rates between the on-site AI and the transported semen were almost 3 percentage points for the FH and 5 percentage points for the SB.

The high pregnancy and foaling rates of the TB as a result of natural cover are in conflict with the results achieved in other breeds. Probably the high value of the breeding stock and foals creates pressures, but also offers opportunities for veterinarians and stud managers to intensively manage, control and treat mares. The AI as such may not be any better than natural mating, but its use requires the use of a veterinarian and as a result, the mares are carefully controlled and examined.

### Foaling rate vs. pregnancy rates

Unfortunately, the original goal to use the pregnancy rate per cycle as the fertility measure was not possible because of frequent missing data. Pregnancy rate per cycle reflects the true fertility of the mare and stallion, whereas the seasonal pregnancy rate can be greatly improved by persistent trying, hard work and good management. Foaling rate, on the other hand, includes the effect of the pregnancy losses. Although this does not necessarily reflect innate fertility, it is the best economical measure. One example for the difference between the fertility measures can be taken from the study of Morris and Allen [9]: the pregnancy rates per cycle were 55.3% for barren mares and 61.4% for foaling mares, the respective seasonal pregnancy rates were 92.4 and 85.6%, and foaling rates 88.8 and 81.2%. The barren mares may be valuable problem mares which get more attention and benefit from veterinary treatments, but also have a risk to lose the pregnancy. In an Australian study, the pregnancy rate per cycle was significantly higher in TB than in SB, but the seasonal pregnancy rates were the same, which was explained by the longer breeding season in SB industry [2]. One can speculate whether the low foaling rates of barren and old mares in Finnish trotters, particularly FH, as compared with those of TB could be explained by deficient veterinary care and reluctance of owners to invest money in the treatments. In the intensive and professional TB industry, veterinarians are an integral part of the business, but in Finland this is not as self-explanatory.

Another example can be taken from the use of frozen semen. There per cycle pregnancy rates are typically low (20-50%), but the seasonal pregnancy rates are close to fresh semen AI (60-90%) [23]. In our data, the foaling rate after frozen semen AI was 64%, which was 6 percentage points lower than for transported semen, but presumably the pregnancy rates per cycle would have differed more. Pregnancy rate per cycle is a more sensitive indicator when the efficiency and economy of breeding is evaluated.

### Sources of error

Our results are prone to some errors due to several steps and several people handling the data starting from the level of accuracy among the stallion holders providing the first information.

It is also possible that some stallion owners purposely left some of the non-pregnant mares out of the book in an attempt to get better pregnancy results for their stallion. Moreover, not all breedings are necessarily reported, because stallion managers might not be aware of the purpose of reporting matings in all heats, not just the last heat of the season.

## Conclusions

For the future, it is very important that the data collection is precise, reliable and as inclusive as possible. This means that all matings in all cycles should be recorded. As the foaling rate is not a very sensitive indicator for fertility and the pregnancy rate per cycle or per first mating would be much better, their use should be made possible. Furthermore, the stud farms should give the breeding reports in real time or at least once a month to avoid the possibility of leaving out data on the non-successful matings.

Precise, accurate and timely breeding data are necessary to detect the possible changes in fertility and breeding efficiency. For this reason, the horse breeding industry needs to be informed in good time about the possible threats to the efficiency of horse breeding.

The descriptive results of this study suggest that factors such as age and type of the mare as well as mating type all have important effects on foaling rates. Moreover, structural differences exist between the two horse breed populations which may explain the differences in foaling rates. Structural differences in the mare populations have taken place during the study period and they are likely to explain the declining foaling rates. A more in-depth statistical multivariable analysis of the data considering the hierarchy of the breeding structure would reveal the relative significance of the different variables.

## Competing interests

The authors declare that they have no competing interests.

## Authors' contributions

KN imported, checked, corrected and analysed the material and drafted the figures. A-M V instructed and supervised the description and statistical analysis of the data and participated in the revision of the manuscript. TR actively participated in the revision and writing of the manuscript. TP represented the organisation that owned the data and was actively involved in the planning of the research and made comments to the text. TK was the initiator and leader of the project and had the major responsibility for writing and finalizing the manuscript. All authors read and approved the final manuscript.

## Authors' information

KN is the co-owner and chief veterinarian of the largest stud farm in Finland concentrating mainly on SB breeding. She is enrolled in a PhD programme at the University of Helsinki. A-M V, Ph.D., is a university lecturer in veterinary epidemiology at the University of Helsinki. TR, Ph.D., is a researcher in equine reproduction in the Equine Research of MTT Agrifood Research Finland. TP, MSc, is responsible for the animal breeding department in Suomen Hippos, the central horse organisation in Finland. TK, Ph.D., is professor in animal reproduction at the University of Helsinki working mainly with equine reproduction.

## References

[B1] Langlois B Blouin C Statistical analysis of some factors affecting the number of horse births in France Reprod Nutr Dev 2004 44 583 595 10.1051/rnd:2004055 15762302

[B2] Reproductive efficiency of horses in Australia http://www.gvequine.com.au/breeding_efficiency.htm 9.1.2009

[B3] Laing JA Leech FB The frequency of infertility in Thoroughbred mares J Reprod Fertil 1975 Suppl 23 307 310 1060795

[B4] Osborne VE Factors influencing foaled percentages in Australian mares J Reprod Fertil 1975 Suppl 23 477 483 1060827

[B5] Sullivan JJ Turner PC Self LC Gutteridge HB Bartlett DE Survey of reproductive efficiency in the Quarter-horse and Thoroughbred J Reprod Fertil 1975 Suppl 23 315 318 1060797

[B6] Sanderson MW Allen WR Huntigton T Reproductive efficiency of Thoroughbred mares in the United Kingdom 9 Proc Bain Fallon Memorial Lectures, AEVA, Sydney 31 41

[B7] Brück I Anderson GA Hyland JH Reproductive performance of thoroughbred mares on six commercial stud farms Aust Vet J 1993 70 299 303 10.1111/j.1751-0813.1993.tb07979.x 8216096

[B8] Hearn P Bonnett B Samper J Factors influencing pregnancy and pregnancy loss on one thoroughbred farm39th Ann Conv Am Assoc Equine Pract 1993 161 163

[B9] Morris LHA Allen WR Reproductive efficiency of intensively managed Thoroughbred mares in Newmarket Equine Vet J 2002 34 51 60 10.2746/042516402776181222 11822372

[B10] Hemberg E Lundeheim N Einarsson S Reproductive performance of Thoroughbred mares in Sweden Reprod Dom Anim 2004 39 81 85 10.1111/j.1439-0531.2004.00482.x 15065988

[B11] Allen WR Brown L Wright M Wilsher S Reproductive efficiency of Flatrace and National Hunt Thoroughbred mares and stallions in England Equine vet J 2007 39 438 445 10.2746/042516407X1737581 17910269

[B12] Bosh KA Powell D Shelton B Zent W Reproductive performance measures among Thoroughbred mares in central Kentucky, during the 2004 mating season Equine vet J 2009 41 883 888 10.2746/042516409X456068 20383986

[B13] Merkt H Jacobs K-O Klug E Aukes E An analysis of stallion fertility rates (foals born alive) from the breeding documents of the Landgestüt Celle over a 158-year period J Reprod Fertil 1979 Suppl 27 73 77 383990

[B14] Davies Morel MCG Gunnarson V A survey of the fertility of Icelandic stallions Anim Reprod Sci 2000 64 49 64 10.1016/S0378-4320(00)00192-5 11078966

[B15] Keiper R Houpt K Reproduction in feral horses: An eight-year study Am J Vet Res 1984 45 991 995 6732036

[B16] Hutton CA Meacham TN Reproductive efficiency on fourteen horse farms J Anim Sci 1968 27 434 438 5646343 10.2527/jas1968.272434x

[B17] Katila T Effects of hormone treatments, season, age and type of mares on ovulation, twinning and pregnancy rates of mares inseminated with fresh and frozen semen Pferdeheilkunde 2003 19 619 624

[B18] Jalostustilastot Suomen Hippos 2008 http://www.hippos.fi/hippos/jalostus_ja_kasvatus/Jalostustilastot.php 24.4.2009

[B19] Chevalier-Clément F Pregnancy loss in the mare Anim Reprod Sci 1989 20 231 244 10.1016/0378-4320(89)90088-2

[B20] Carnevale EM Ginther OJ Relationship of age to uterine function and reproductive efficiency in the mare Theriogenology 1992 37 231 246 10.1016/0093-691X(92)90108-4 16727108

[B21] McDowell KJ Powell DG Baker CB Effect of book size and age of mare and stallion on foaling rates in thoroughbred mares J Equine Vet Sci 1992 1 364 367 10.1016/S0737-0806(06)81363-8

[B22] Samper JC Management and fertility of mares bred with frozen semen Anim Reprod Sci 2001 68 219 228 10.1016/S0378-4320(01)00158-0 11744266

[B23] Barbacini S Marchi V Zavaglia G Equine frozen semen: results obtained in Italy during the 1994-1997 period Equine vet Educ 1999 11 109 112 10.1111/j.2042-3292.1999.tb00930.x

